# Leisure-Time Physical Activity in People With Spinal Cord Injury—Predictors of Exercise Guideline Adherence

**DOI:** 10.3389/ijph.2022.1605235

**Published:** 2022-12-12

**Authors:** Paul K. Watson, Mohit Arora, James W. Middleton, Camila Quel de Oliveira, Robert Heard, Andrew Nunn, Timothy Geraghty, Ruth Marshall, Glen M. Davis

**Affiliations:** ^1^ Discipline of Exercise and Sport Sciences, Sydney School of Health Sciences, Faculty of Medicine and Health, The University of Sydney, Sydney, NSW, Australia; ^2^ Northern Sydney Local Health District, John Walsh Centre for Rehabilitation Research, The Kolling Institute, Sydney, NSW, Australia; ^3^ Translational Research Collective, Faculty of Medicine and Health, The University of Sydney, Sydney, NSW, Australia; ^4^ Department of Physiotherapy, Graduate School of Health, Faculty of Health, University of Technology Sydney, Ultimo, NSW, Australia; ^5^ Discipline of Behavioural and Social Sciences in Health, Faculty of Medicine and Health, Sydney School of Health Sciences, The University of Sydney, Sydney, NSW, Australia; ^6^ Victorian Spinal Cord Service, Austin Health, Heidelberg, VIC, Australia; ^7^ Queensland Spinal Cord Injuries Service, Division of Rehabilitation, Princess Alexandra Hospital, Brisbane, QLD, Australia; ^8^ The Hopkins Centre, Griffith University, Brisbane, QLD, Australia; ^9^ South Australian Spinal Cord Injury Service, Central Adelaide Local Health Network, Adelaide, SA, Australia; ^10^ Faculty of Health and Medical Sciences, University of Adelaide, Adelaide, SA, Australia

**Keywords:** spinal cord injury, exercise, physical activity, community survey, Australia, physical activity guidelines, leisure time physical activity

## Abstract

**Objectives:** This study described leisure-time physical activity (LTPA) for people in Australia with spinal cord injury (SCI) and whether certain sociodemographic and psychosocial variables might be associated with LTPA uptake and guidelines adherence.

**Methods:** The Physical Activity Scale for Individuals with a Physical Disability was used to measure the intensity and volume of LTPA of 1,579 individuals with SCI. Summary statistics were calculated for LTPA guidelines adherence. Analyses included regression modelling.

**Results:** Of the 1,579 participants, 58% performed LTPA and 13% adhered to recommended guidelines for weekly LTPA. There was an association with being an “exerciser” based on the time since injury (OR = 1.02 [95% 1.01–1.03]), a traumatic injury (OR = 1.53 [95% CI 1.13–2.08]) and a higher self-rating of health (OR = 1.10 [95% CI 0.95–1.27]). Where LTPA guidelines were met, adherence was most related to a traumatic injury (OR = 1.75 [95% CI 1.02–3.02]) and being unemployed (OR = 1.53 [95% CI 1.03–2.25]).

**Conclusion:** Of those who performed LTPA with SCI, one in four met population-specific LTPA guidelines. Sociodemographic variables were moderately associated with being an “exerciser” or LTPA “guideline-adherent.”

## Introduction

Spinal cord injury (SCI) is a complex and often severe neurological disorder resulting in significant neuromuscular impairment with loss of movement, physical deconditioning, autonomic disruption to internal organs, and chronic disability. As a result, the risk of cardiometabolic syndrome increases, and cardiovascular-related morbidity and mortality are hastened [1]. In addition, because of reduced physical capacity, the energy expenditure in this population is generally low [2], and barriers to engaging in leisure-time physical activity (LTPA) are numerous [3].

Pneumonia and other pulmonary complications are common causes of illness after SCI, and cardiovascular disease is the most common cause of death [4]. People with SCI are 5% more likely to develop anxiety, 20% more likely to develop depressive disorders and 15% more likely to develop psychological multimorbidity than people without SCI [4]. Whilst a physically active lifestyle and increased engagement in LTPA may reduce the risk of preventable disease associated with SCI [5], an appropriate intensity of LTPA is needed to overcome functional limitations, reduce the risk of development of physiological and psychological comorbidities, and increase daily energy expenditure [6–8].

The first SCI-specific LTPA guidelines were published in 2011 [9]. These guidelines recommended that individuals with SCI should perform 20 min of moderate-to-vigorous intensity aerobic exercise and three sets of moderate-to-vigorous strength-training activities (for each major functioning muscle group) twice weekly to augment cardiorespiratory fitness. In 2018, these guidelines were updated, and the authors recommended that for improved cardiometabolic health, adults with SCI should engage in 30 min of moderate-to-vigorous intensity aerobic exercise three times per week; strength training recommendations were unchanged [10]. These recommendations presented an alternative to the current World Health Organisation (WHO) guidelines of 150–300 min per week of accumulated moderate-intensity or 75–150 min per week of accumulated vigorous-intensity aerobic exercise, with muscle-strengthening activities on 2 days per week [11]. However, the WHO guidelines were not specifically tailored to the SCI population. Whilst WHO recommendations could be applied to individuals with a disability, adjustments might be required based on their low exercise capacity and specific health risks or medical limitations. Thus, the deployment of SCI-specific LTPA guidelines was a positive step to reduce all-cause morbidity and mortality in this sedentary population. Unfortunately, however, these SCI-specific recommendations are generally not met [12, 13], and the trend for poor exercise engagement is pervasive. For example, research in Thailand [14], Canada [15], and Germany [16] has reported that 49%–50% of adults with SCI undertake no weekly LTPA.

The International Spinal Cord Injury (InSCI) Community Survey was initiated in response to the International Perspectives on Spinal Cord Injury recommendations, and systematically collected data on approximately 12,500 adults with traumatic or non-traumatic SCI, with Australia being one of the 22 participating countries [17]. Data for the current study were obtained from the Australian cohort of the InSCI (known as the Aus-InSCI survey [18]), providing an opportunity to understand the relationship of LTPA uptake with factors such as income, level of education, marital status, level and completeness of injury, and feelings of energy, general health, and life satisfaction.

Previous research on people with SCI has suggested an association between LTPA volume and sociodemographic and psychosocial traits such as gender, time since injury, severity of injury and feelings of self-worth and control [8, 19]. With the biopsychosocial benefits of LTPA clearly established [20, 21], researchers, practitioners and policymakers must understand the current state of LTPA uptake within the SCI community and examine what can facilitate a beneficial volume of LTPA. Therefore, this study sought to investigate the proportion of individuals with SCI adhering to non-disabled and population-specific LTPA guidelines, to analyse whether individuals with specific injury and lifestyle characteristics were likely to exercise and, if so, to follow LTPA recommendations.

## Methods

### Design

This study was a retrospective analysis of the data collected in the Aus-InSCI community survey, which formed part of the global cross-sectional InSCI Community study. Study design and procedures for both InSCI and Aus-InSCI surveys have been described previously [18, 22, 23]. The Aus-InSCI community survey was approved by the Northern Sydney Local Health District HREC (HREC/16/HAWKE/495) and the Australian Institute of Health and Welfare Ethics Committee (EO2017/1/341).

### Participants

Participants included individuals aged 18 years or over who were residing in the community, could fill in the questionnaire in English and had sustained either a traumatic or non-traumatic SCI disease or disorder at least 12 months prior.

### Data Linkage

An anonymised master dataset was created by combining data from 11 databases across nine data custodians, including state-wide SCI clinical services, not-for-profit consumer organisations, and a government insurance agency across four Australian states. Databases were linked, de-duplicated and cleaned by an external data linkage facility, the Population Health Research Network - Centre for Data Linkage at Curtin University in Western Australia, before being forwarded to the Australian Institute of Health and Welfare for linkage to the National Death Index to remove records of deceased individuals. Eligible individuals were invited to participate by each data custodian, with two reminders sent to individuals who had not responded three and 6 months after the initial invitation. Surveys were completed *via* hardcopy or electronically between March 2018 and January 2019.

### Measures

The InSCI data model [23] was based on the International Classification of Functioning, Disability and Health Core Sets for SCI and Rehabilitation, with 47 categories covered by the InSCI questionnaire. The Aus-InSCI questionnaire [18, 22], written in English, contained the international module (consisting of 125 questions) and a national module with 68 additional questions, all of which took 45–60 min to complete. Responses from the international module included in this study were categorised into Personal Information and Injury Characteristics, Quality of Life and Psychosocial Attributes, and Leisure-time Physical Activity domains.

#### Leisure-Time Physical Activity

Physical activity volume was gathered using a modified version of The Physical Activity Scale for Individuals with Physical Disabilities (PASIPD) [24]. The PASIPD gathers and encodes information concerning physical activities performed by the individual for both exercise and lifestyle activities. The survey tool instructs participants to recall in the previous 7 days how many days per week (days/wk) they engaged in a particular activity (never, seldom (1–2 days/wk); sometimes (3–4 days/wk); or often (5–7 days/wk)), and for how many hours each day they participated in it (<1 h, 1–2 h, 2–4 h, or, >4 h). However, in the Aus-InSCI survey, these responses were removed and replaced with a free-text space so participants could input the number of days per week and minutes per day they performed each intensity of LTPA. In this study, only the questions listed under Physical Activity in Supplementary Material SA were included to provide a value of total LTPA (in minutes per week) since they related directly to exercise/LTPA. Other activities related to daily living (e.g., gardening, home repairs, occupation, caring for others) or ambulation, were not included.

#### LTPA Guidelines

The LTPA data was used to ascertain the percentage of participants meeting the SCI LTPA guidelines [9, 10] or the WHO moderate or vigorous intensity LTPA guidelines [11]. These guidelines are:

(1) SCI LTPA guidelines: 20 min twice per week of moderate-to-vigorous physical activity, and strength training activities for each major muscle group on 2 days per week.
(2) WHO guideline 1 (WHO LTPA_Mod_): 150 min of accumulated moderate-intensity physical activity per week and muscle strengthening activities on 2 days per week.
(3) WHO guideline 2 (WHO LTPA_Vig_): 75 min of accumulated vigorous-intensity physical activity per week and muscle strengthening activities on 2 days per week.

#### Personal Information and Injury Characteristics

Sociodemographic data, including age, gender, marital status, pre- and post-injury employment status, education level and weekly household income, were analysed, along with information about neurological characteristics (i.e., level and completeness of injury) and cause of injury (i.e., traumatic or non-traumatic). Continuous age and time since injury data were categorised into ranges as recommended by DeVivo et al. [25]. The data in the categories years of education before SCI and years of education after SCI were converted into an ordinal format, and ranges were aligned with the recommendations for time since injury from DeVivo et al. up to 15 years, with a final category of 16+ years.

#### Quality of Life and Psychosocial Attributes

The Vitality and Mental Health domains of the 36-item Short-Form Health Survey (SF-36) [26] were included in this study, utilising the RAND method [27] for individual scoring. The level of independence was assessed using the modified version of the self-reported Spinal Cord Independence Measure (SCIM-SR) [28]. Perceived self-efficacy was assessed by two general items from the General Self-Efficacy Scale (GSES) [29] and two SCI-specific items from the Moorong Self-Efficacy Scale (MSES) [30], with scores summed (max score of 20). Quality of life (QOL) was assessed by a combined score of one global QOL question and five questions from the Abbreviated World Health Organisation QOL questionnaire [31] for health, physical and psychological wellbeing, social relationships, and the environment. In addition, two questions rated the current state of general health and changed health over the preceding 12 months.

### Analysis

#### “Exerciser” and “Guideline-Adherent” Cohort Definitions

“Exercisers” were defined as individuals who reported performing one or more minutes of LTPA per week.

“Non-exercisers” were classified as individuals who recorded 0 minutes of LTPA per week. Missing values for total minutes of light, moderate, or vigorous-intensity, and strength LTPA were taken to mean none.

A person was classified as a “guideline-adherent” if they had met the requirements of the SCI-specific guidelines or one of the WHO guidelines, but as “non-adherent” if not.

The modified PASIPD did not collect specific set and repetition data for strength exercises, nor what specific strengthening exercises were performed. Thus, participants were considered to have met the strength physical activity requirement if they reported strength exercises on two more days per week for a total of 40 min or more. Methods to ascertain guideline compliance using survey data like this have been used previously within this population and for these guidelines [12].

#### Measures of Association

Due to the anonymous nature of the Aus-InSCI survey, it was not possible to check response quality.

Independent variables were dichotomised (where necessary) for effect size and regression analyses. ANOVA was used to investigate differences between independent variables and LTPA for “exercisers” vs. “non-exercisers,” and again for “guideline-adherents” vs. “non-adherents.” Comparison and significance were examined using measures of central tendency.

Bivariate screening using Pearson’s Chi-square and ANOVA F statistic tested for significant differences between “exercisers” vs. “non-exercisers” and “guideline-adherents” vs. “non-adherents” for each independent variable (Supplementary Material SB). Regression analysis was performed twice. The first analysis used the entire sample to model predictors of “exerciser” vs. “non-exercisers.” The second analysis used only the “exercisers” to model predictors of “guideline-adherents” vs. “non-adherents.” Bivariate backward stepwise logistic regressions were conducted on the independent variables as a screening procedure to identify potential predictor variables for each comparison. Subsequently, standard multiple logistic regressions were performed on the candidate variables previously identified. Variables that achieved a *p* ≤ 0.20 [32] significance level in the Chi-square and ANOVA screening analyses were used in the regression modelling.

In all final models, predictors with a *p*-value <0.05 are reported as statistically significant. The Tukey HSD test was chosen for follow-up of all significant omnibus ANOVA analyses with more than two levels of the independent variable. All statistical analyses were performed using the SPSS v27 software for Windows.

## Results

### Cohort Characteristics

The majority of the 1,579 participants were male (73%), married (50%) and had an incomplete spinal cord lesion (37% paraplegia, 30% tetraplegia). Participants with complete tetraplegia comprised approximately 9% of the sample, with the remainder (24%) having complete paraplegia. Most (82%) had a traumatic aetiology of injury. The mean (SD) age of participants was 57 (14) years, with a mean (SD) time since their injury of 17 (14) years. Sociodemographic characteristics (as shown in Supplementary Material SC) were similar between “exercisers,” “non-exercisers,” “guideline-adherents” and “guideline non-adherents,” except that “guideline-adherents” had a 10% lower employment rate than the other groups.

“Guideline-adherents” reported a 10% higher level of vitality than “non-adherents,” and “exercisers” reported an 8% higher level of vitality than ‘non-exercisers’ (*p* < 0.05). “Guideline-adherents” reported the highest scores in health satisfaction, general health and quality of life (satisfaction) and personal factors (confidence). Vitality ratings were identical between “exercisers” and “non-exercisers” (Supplementary Material SD).

### Leisure-Time Physical Activity and LTPA Guidelines

Of the participants’ responses, 669 (42%) reported having done no LTPA in the last 7 days (“non-exercisers”), whereas 910 (58%) reported performing one or more minutes per week of LTPA (“exercisers”).

Of the complete cohort, 5.8% met or exceeded the WHO LTPA_Mod_ and 7.5% met or exceeded the WHO LTPA_Vig_ guidelines. For the SCI population-specific guidelines, 9.9% met or exceeded the SCI LTPA guidelines of 40 min of moderate-intensity aerobic exercise plus two strength-training bouts per week. “Exercisers” (*n* = 910) had an adherence of 10% to the WHO LTPA_Mod_, 13% for the WHO LTPA_Vig_, and 17.1% for the SCI LTPA recommendations, as shown in Table 1.

**TABLE 1:** Leisure-time physical activity guidelines adherence (Australia, 2022).

LTPA guidelines	All participants (*n* = 1,579)		Exercisers (*n* = 910)	
	Number met (%)	Number not met (%)	Number met (%)	Number not met (%)
40 min of Moderate Aerobic Exercise + 2 days of Strength Exercise per week (SCI LTPA guidelines) [10]	156 (9.9)^†^	1,423 (90.1)	156 (17.1)^Δ^	754 (82.9)
150 min of Moderate Aerobic Exercise + 2 days of Strength Exercise per week (WHO_Mod_) [11]	91 (5.8)^†^	1,488 (94.2)	91 (10)^Δ^	819 (90)
75 min of Vigorous Aerobic Exercise + 2 days of Strength Exercise per week (WHO_Vig_) [11]	118 (7.5)^†^	1,461 (92.5)	118 (13)^Δ^	792 (87)

^†^ *p* < 0.05 Chi-square test between “all participants.”

^Δ^ *p* < 0.05 Chi-square test between “exercisers.”

Abbreviations: LTPA, leisure-time physical activity; SCI, spinal cord injury; WHO, World Health Organisation.

**FIGURE 1:**
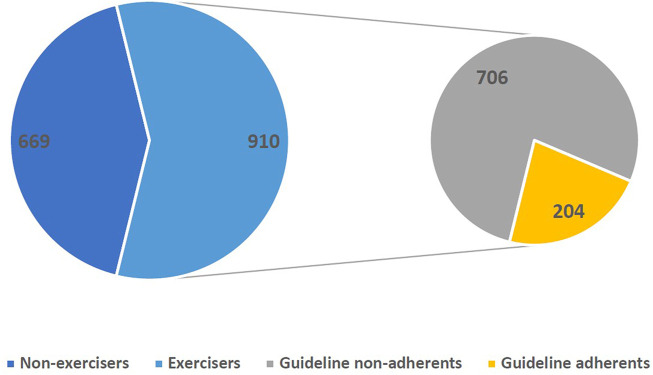
Proportions of “Exercisers” and Guideline adherents (Australia, 2022).

Figure 1 shows that 204 survey respondents of the entire sample met at least one of the three LTPA guidelines. These 204 people constituted 12.9% of the total cohort and 22% of the “exerciser” group. Of those who were “guideline-adherent,” 50 respondents (25%) met the SCI LTPA guidelines, 36 (18%) met the LTPA_Mod_, and 48 (24%) achieved the WHO LTPA_Vig_. Fifteen individuals (7%) achieved the SCI-specific guidelines and the WHO LTPA_Vig_, and 55 persons (27%) met the SCI LTPA, WHO LTPA_Mod_ and WHO LTPA_Vig_ guidelines.

**FIGURE 2:**
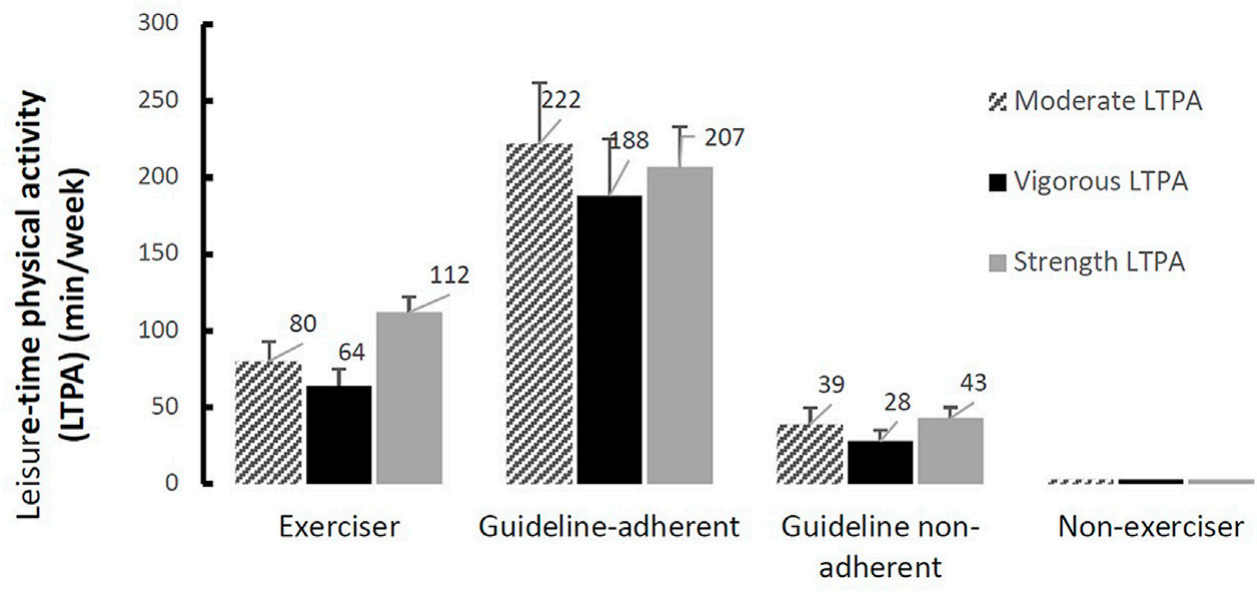
Mean minutes of leisure-time physical activity with 95% confidence intervals (Australia, 2022).

Individuals who adhered to either guidelines (*n* = 204) for both moderate and vigorous-intensity LTPA performed between two and three times as much total LTPA as all individuals who exercised (*n* = 910). Figure 2 portrays the mean minutes of total LTPA for “exercisers,” “non-exercisers,” “guideline-adherents” and “guideline non-adherents.”

### Association of Exercising and Adhering to Guidelines With Sociodemographic, Self-Efficacy, and Other Variables

Age, time since injury, cause of injury, household income, education level, and years of education (pre-SCI) were all related to whether an individual might be an “exerciser” or “non-exerciser.” For those who undertook LTPA, gender, cause of injury, household income and current employment status were associated with a person achieving guideline recommendations of exercise. All psychosocial variables influenced a participant being an “exerciser” and “guideline-adherent,” except for QOL-rating for “guideline-adherents” (Tables 2, 3).

**TABLE 2:** Odds ratios between sociodemographic and psychosocial variables with “Exercisers” (*n* = 910) and “Non-Exercisers” (*n* = 669) (Australia, 2022).

Group these figures apply to	Exercisers	
Group referenced against	Non-exercisers	
**Statistic**		
Model significance	*X* ^2^ = 52.114, *p* < 0.001	*X* ^2^ = 58.701, *p* < 0.001
Strength of prediction	Cox and Snell *R* ^2^ = 0.046	Cox and Snell *R* ^2^ = 0.039
	Nagelkerke *R* ^2^ = 0.062	Nagelkerke *R* ^2^ = 0.053
Total cases classified correctly	62.8%	62.8%
**OR [95% CI]**		
Time since Injury	1.02 [1.01–1.03] *p* < 0.001*	
Cause of Injury (traumatic)^a^	1.53 [1.13–2.08] *p* = 0.023*	
Household Income	0.95 [0.91–1.00] *p* = 0.045*	
Education Level	0.87 [0.81–0.93] *p* < 0.001*	
Vitality		0.99 [0.99–1.00] *p* = 0.012*
Health Rating		1.38 [1.18–1.61] *p* = 0.001*
Health Satisfaction		1.10 [0.95–1.27] *p* = 0.006*

^a^ Reference: Non-traumatic.

*p ≤ 0.05.

Regression models were conducted twice: once for sociodemographic variables and once for psychosocial variables.

Abbreviations: OR, odd ratios; CI, confidence interval.

**TABLE 3:** Odds ratios between sociodemographic and psychosocial variables with “Guideline adherents” (*n* = 204) vs. “Non-adherents” (*n* = 706) (Australia, 2022).

Group these figures apply to	Guideline-adherent	
Group referenced against	Non-adherent	
**Statistic**		
Model significance	*X* ^2^ = 9.809, *p* = 0.007	*X* ^2^ = 35.497, *p* < 0.001
Strength of prediction	Cox and Snell *R* ^2^ = 0.012	Cox and Snell *R* ^2^ = 0.039
	Nagelkerke *R* ^2^ = 0.018	Nagelkerke *R* ^2^ = 0.060
Total cases classified correctly	87.2%	86.5%
**OR [95% CI]**		
Cause of Injury (traumatic)^a^	1.75 [1.02–3.02] *p* = 0.044*	
Employment status (not working)^b^	1.53 [1.03–2.25] *p* = 0.033*	
Vitality		1.02 [1.01–1.02] *p* = 0.002*
Health Rating		0.84 [0.61–0.91] *p* = 0.004*

^a^ Reference: Non-traumatic.

^b^ Reference: Currently employed.

Regression models were conducted twice: once for sociodemographic variables and once for psychosocial variables.

*p < 0.05.

Abbreviations: OR, odd ratios; CI, confidence interval.

The sociodemographic and psychosocial regression models correctly identified “exercisers” 63% of the time, and both “exerciser” and “guideline-adherent” logistic regression models were statistically significant. However, as seen in Table 2, odds ratios were generally quite weak. For example, individuals with a traumatic SCI were approximately 1.5 times more likely (OR = 1.53 [95% CI 1.13–2.08, *p* = 0.023]) to be an “exerciser,” and as time since injury progressed, there was a slightly increased chance a person would be a “exerciser” (OR = 1.02 [95% CI 1.01–1.03] *p* < 0.001).

The logistic regression model for guideline-adherence correctly identified “guideline-adherents” 87% of the time (Table 3). “Exercisers” with traumatic SCI were 1.8 times more likely to achieve guideline levels of LTPA (OR = 1.75 [95% CI 1.02–3.02] *p* = 0.044), and “exercisers” who were unemployed were 1.5 times more likely to be adherent to LTPA guideline recommendations (OR = 1.525 [1.03–2.25] *p* = 0.033).

## Discussion

This study sought to investigate LTPA guideline adherence in the Australian population with SCI and whether sociodemographic or psychosocial variables were associated with a likelihood of an individual undertaking LTPA and achieving LTPA guideline recommendations. Gender, age, cause of injury, time since injury, marital status, household income, education level, employment status, and self-rated vitality and health had mild-to-moderate associations with LTPA uptake and volume.

The characteristics of an “exerciser” and “guideline-adherent” in this study were not unusual compared to previously-published research [15, 19, 20]. Generally, males performed more LTPA than females, younger individuals performed more LTPA than older individuals, and those with a traumatic SCI reported more physical activity than those with a non-traumatic SCI. These trends appeared consistent for all subgroups within the current study.

### LTPA Guidelines

Fifty-five percent of Australian adults do not meet physical activities guidelines, and this increases with age [33, 34]. Adults with a diagnosed disability are even less likely to meet exercise guidelines, with 72% failing to achieve recommended minimum LTPA levels [33, 34]. The data in this study highlighted that only 12%–13% of Australians with SCI in the sample met any of the recommended LTPA guidelines, and 42% reported having an entirely sedentary lifestyle. It was an important finding from this national survey that 87% of the population with SCI met neither the SCI-specific LTPA nor the WHO LTPA guidelines—32% more than the Australian adult non-disabled population and 15% more than the broader Australian disability community (measured using the WHO LTPA guidelines) [33, 34].

Studies investigating whether individuals with SCI meet the WHO or population-specific LTPA guidelines are emerging. Previous research in Switzerland [19] reported that 49% of its SCI population adhered to guideline levels of LTPA, and only 19% of their study sample was physically inactive. A study in Canada [15] observed that only 12% of participants met LTPA guidelines, and 44% reported 0 min of LTPA. Similar levels of LTPA inactivity in the German SCI population have been reported by Annekan et al. [16], with rates of 52% of the population involved in physical activity and 49% not. Compared to the general Australian populace and the Australian disability community, people with SCI in Australia have some of the lowest weekly levels of LTPA [33, 34].

### Association of Exercising and Adhering to Guidelines With Sociodemographic, Self-Efficacy, and Other Variables

While there is substantial research published on correlates of exercise behaviour in the general community [35–37], there is less in SCI-specific populations [12, 15] and little on guideline-adherence in this cohort [12, 13]. Considering the lack of attention to physical activity and the low guideline-adherence rate in people with SCI (lower than the broader disability exercise adherence rate), substantially increased focus from researchers and clinicians to improve LTPA uptake is vital. This study identified that age, cause of injury, time since injury, marital status, income and education level are significantly associated with who is likely to be an “exerciser” vs. a “non-exerciser.” A higher perception of individual health appears to be one the stronger predictors of an “exerciser” but we concede that a higher self-reported rating of health may be the result of exercising, rather than its cause. Drawing inferences from this finding should be carefully done considering this uncertainty of “cause and effect.”

Whilst there were notable differences between genders for LTPA volume (“guideline-adherent” and “non-adherent”), the logistic regression did not reveal gender as a significant predictor of LTPA uptake (“exerciser” and “non-exerciser”). This finding is supported by previous studies that found women with SCI had lower probabilities of performing enough LTPA to fulfil guideline requirements and generally reported reduced LTPA levels [12, 15, 19]. Our results suggested that gender does not seem to influence LTPA commencement, but rather the amount of LTPA that will be undertaken and the intensity at which it will be performed. Therefore, future interventions should examine methods to improve weekly LTPA intensity and duration for women with SCI.

Our study found that there were differences between age and LTPA uptake (but not volume). Previous research [19] investigating age has highlighted that people with SCI aged 71 and older had the highest probabilities of being physically inactive. Furthermore, those 31 to 50-year-olds and 50 to 71-year-olds had slightly higher odds of being physically idle compared to 17 to 30-year-olds. These findings highlight the importance of encouraging individuals to embed LTPA into their daily lifestyle as early as possible, knowing that once commenced, the volume doesn’t seem to fluctuate much with increasing age. In addition, a perceived lack of benefit, lack of energy, lack of fitness and poor health have been reported as significant barriers to LTPA uptake in older individuals [38].

Time post-injury strongly correlates with this population’s exercise behaviour [15, 19]. Although the Aus-InSCI study found that likelihood of LTPA uptake decreased as time after injury increased, overall chronicity was a weak predictor of exercise uptake. Previous research has revealed that disability and the reduced physical capacity associated with secondary health sequelae are common after long-standing SCI [38]. Moreover, recently injured people are more likely to receive encouragement and support for LTPA than those whose injuries occurred many years previously [39]. These findings highlight the importance of encouragement and facilitation of positive exercise behaviours as early as possible after injury.

Individuals with traumatic SCI were significantly more likely to undertake LTPA and were considerably more likely to achieve recommended levels of LTPA, highlighting the importance to providing exercise opportunities and support to those injured non-traumatically. Research in epidemiology of non-traumatic SCI has shown that it is significantly more associated with older individuals [40] who, as this and previous [19] research has shown, are already less likely to uptake LTPA than younger individuals. Despite the InSCI study not investigating differences in injury level as some prior surveys have done, our findings were (still) consistent with previous research whereby people with motor-incomplete injuries performed significantly more LTPA than those with motor-complete injuries [15]. In general, the more assistance the individual requires with ambulation, the lower the levels of weekly LTPA [15]. Thus, LTPA services should ensure that there are adequate exercise capacities for all individuals, regardless of the level of SCI, in an effort to facilitate LTPA in people with greater severities of injury.

Employment status had one of the strongest associations with guideline adherence in the current study. Individuals who were not employed were 1.5 times more likely to achieve guideline levels of LTPA than those in employment. In contrast, previous research has highlighted that employed individuals with SCI performed larger volumes of LTPA [41]. So, this may indicate that in the current Australian cohort, employment did not influence the uptake, as much as the volume, of weekly LTPA. Nevertheless, attention should be given to facilitating higher LTPA levels for employed “exercisers,” and employment should be encouraged for its reported benefits [42], confident in the knowledge that uptake of paid work won’t result in a reduced total average LTPA volume.

Fatigue contributes to the reduction in health-related QOL and is common after SCI, with 52–57% of people living in the community reporting it as a secondary impairment [43]. In addition, studies have shown that depression and sedating medications are common after SCI and have a propagating effect on fatigue [44]. In contrast, exercise has a positive impact on mental illness and has the potential to improve energy levels [45]. In this study, “exercisers” and “guideline-adherents” reported higher vitality levels than “non-exercisers” and “non-adherents.” These findings highlight the importance of identifying modifiable causes of fatigue and promoting LTPA in this population to reduce it, improve physiological energy capacity and thus ultimately increase LTPA uptake and adherence.

### Strengths and Limitations

This study’s large population-based sample offered insights and baseline lifestyle data about current LTPA experiences and behaviours of people with SCI and what influences them. The findings are essential to inform service providers and policymakers about improving physical and psychosocial health and wellbeing. However, this study had some limitations that constrain the findings.

First, there were large amounts of missing data in a portion of the modified PASIPD instrument. The missing data suggests some (approximately 200) participants under-reported the actual volume of LTPA undertaken. Our study also found that 3.4% (53 participants) of the sample reported what seemed to be excessively high levels of LTPA for everyday daily life (e.g., 5–8 h/day of “exercise”), which represents greater than +3 standard deviations above the mean total LTPA of 1,330 min/wk. Although this was a confounding limitation of our study, it may reflect that in a large-scale survey methodology, some people either over-report their daily LTPA or misunderstand that wheelchair ambulation for daily activities is not LTPA.

Second, all LTPA guidelines advise strength training for major muscle groups twice per week, but unlike the aerobic portion of the guidelines, they don’t specify a minimum duration (time). This survey reported the number of days and minutes per week a participant undertook strength training. We decided that 40 min of strength training spread across two or more days would be sufficient to cover the guideline requirement for each participant, and methods similar to this have been used previously [12] to discern guidelines adherence using the SCI-specific LTPA guidelines. However, it is possible (although unlikely) that some participants could achieve the strength training requirements of LTPA in less time across two or more days. The number of participants who reported two or more days per week but less than 40 min of strength training and achieved aerobic guidelines was 14. If these 14 (less than 1%) participants did achieve LTPA guidelines, it would increase the percentage of the cohort that met guidelines from 204 (12.9%) to 218 (13.8%).

### Conclusion

Individuals with SCI in Australia have low adherence to LTPA guidelines. A little over half of Australians with SCI engage in any LTPA, and of those who do, only one in every four perform enough LTPA to meet SCI-specific or general LTPA guidelines. Few sociodemographic and psychosocial variables seem to predict LTPA behaviour. Practitioners and policymakers should consider the mechanism of injury, time since injury, employment status and education level, as these factors have at least a moderate influence on facilitating LTPA uptake and volume. Particular attention should be given to facilitating increased LTPA uptake and volume in women, older individuals and people with a non-traumatic SCI.
