# Supplementary material for: Leisure-Time Physical Activity in People With Spinal Cord Injury—Predictors of Exercise Guideline Adherence
Source: Int J Public Health. 2022 Dec 12;67:1605235. doi: 10.3389/ijph.2022.1605235 (PMC9790928; doi:10.3389/ijph.2022.1605235)
Supplement: Supplementary file 1 [file DataSheet1.docx]

**Supplement SA. Questionnaire data from the Australian cohort of the International Spinal Cord Community survey that was analysed in this study (Australia, 2022).**

| **Domain** | **#, Field Label (Variable Field Name)** |
| --- | --- |
| Physical Activity | #242: During the past seven days, how many days did you walk, wheel, (hand) cycle outside your home for reasons other than specifically for exercise (days per week)? (Q155a)  #243: on average, how many minutes per day did you spend walking, wheeling or (hand) cycling outside your home (minutes per day)? (Q155b)  #244: During the last seven days, how many days did you engage in light sport or recreational activities or other similar activities (days per week)? (Q156a)  #245: On average, how many minutes per day did you spend in these light sport or recreational activities (minutes per day)? (Q156b)  #246: During the last seven days, how many days did you engage in moderate sport or recreational activities or other similar activities (days per week)? (Q157a)  #247: On average, how many minutes per day did you spend in these moderate sport or recreational activities (minutes per day)? (Q157b)  #248: During the last seven days, how many days did you engage in strenuous sport or recreational activities or other similar activities (days per week)? (Q158a)  #249: On average, how many minutes per day did you spend in these strenuous sport or recreational activities (minutes per day)? (Q158b)  #250: During the last seven days, how many days did do any exercise specifically to increase muscle strength and endurance (days per week)? (Q159a)  #251: On average, how many minutes per day did you do any exercise specifically to increase muscle strength and endurance (minutes per day)? (Q159b) |
| Personal Information | #9: Sex (Q1)  #16: Marital status (Q4)  #24: Education (Q7a)  #26: Education years before SCI (Q8a)  #27: Education years after SCI (Q8b)  #28: Income household (Q9) |
| Recorded/Derived Data   1. *International Spinal Cord Association, Template for Demographics (ISCoS)* 2. *Swiss Spinal Cord Injury Cohort Study (SwiSCI)* | #294: Age, continuous (age_ak)  #296: Merged level and completeness (lev_comp)  #297: Merged causes of injury (moi)  #298: Time since injury, continuous (doi_cont_ak) |
| Energy and Feeling   1. *Short Form Health Survey (SF-36) Vitality Subscale* | #36: Did you feel full of life? (Q15_sf36)  #40: Did you have a lot of energy? (Q19_sf36)  #42: Did you feel worn out? (Q21_sf36)  #44: Did you feel tired? (Q23_sf36) |
| Independence in Activity of Daily Living   1. *Spinal Cord Independence Measure Self-Report (SCIM-SR)* | #121: Moving around moderate distances (10 to 100m) (Q71 (SCIM-SF Q13)) |
| Work   1. *International Labour Market Integration Assessment for Spinal Cord Injury (ILIAS)* | #124: Did you have a job before SCI? (Q71a)  #136: Are you currently engaged in paid work? (Q76) |
| Personal Factors   1. *The General Self-Efficacy Scale (GSES)* 2. *Moorong Self-Efficacy Scale (MSES)* | #176: How confident are you that you can find the means and ways to get what you want if someone opposes you? (Q108) (GSES Q2)  #177: How confident are you that you could deal efficiently with unexpected events? (Q109) (GSES Q4)  #178: How confident are you that you can maintain contact with people who are important to you? (Q110) (MSES Q9)  #179: How confident are you that you can maintain good health? (Q111) (MSES Q15) |
| Quality of Life and General Health   1. *World Health Organisation Quality of Life-100 abbreviated version (WHOQoL – BREF)* 2. *Short Form Health Survey (SF-36)* | #188: How would you rate your quality of life? (Q118) (*WHOQoL Q1)*  #189: How satisfied are you with your health? (Q119) (*WHOQoL Q2)*  #190: How satisfied are you with your ability to perform your daily living activities? (Q120) (*WHOQoL Q17)*  #191: How satisfied are you with yourself? (Q121) (*WHOQoL Q19)*  #192: How satisfied are you with your personal relationships? (Q122) (*WHOQoL* Q20)  #193: How satisfied are you with your living conditions? (Q123) (*WHOQoL Q23)*  #194: In general, would you say your health is: excellent, very good, good, fair, poor? (Q124) (SF36 Q1)  #195: Compared to one year ago, how would you rate your health in general now: much better, somewhat better, about the same, somewhat worse, much worse? (Q125) (SF36 Q2) |

**Supplement SB. Sociodemographic and psychosocial correlates of exercise behaviour between ‘Exercisers’ and ‘Non-exercisers’, ‘Guideline adherents’ and ‘Non-adherents’ and the different combinations of leisure-time physical activity guideline groups (Australia, 2022).**

|  | **‘Exercisers’ (n=910) and ‘Non-exercisers’ (n=669)** | | **‘Guideline adherents’ (n=204) and ‘Non-adherents’ (n=706)** | |
| --- | --- | --- | --- | --- |
| **Sociodemographic** | **F-test/*X*^2^** | **Significance (p)** | **F-test/*X*^2^** | **Significance (p)** |
| **Gender** | 0.036 | 0.594 | 2.147 | 0.142* |
| **Age** | 6.56 | 0.010** | .963 | 0.963 |
| **Injury level and Completeness** | 2.66 | 0.447 | 1.378 | 0.710 |
| **Time since Injury** | 27.5 | <0.001** | 0.792 | 0.591 |
| **Cause of Injury** | 8.789 | 0.012** | 6.888 | 0.032** |
| **Marriage Status** | 7.96 | 0.093* | 4.14 | 0.387 |
| **Household income** | 25.4 | 0.003** | 16.249 | 0.061* |
| **Employed pre-SCI** | 0.582 | 0.446 | 0.258 | 0.611 |
| **Employed currently** | 0.282 | 0.595 | 6.815 | 0.009** |
| **Education Level** | 46.4 | <0.001** | 1.842 | 0.968 |
| **Education years pre-SCI** | 8.62 | 0.003** | 1.482 | 0.321 |
| **Education years post-SCI** | 1.59 | 0.208 | 0.807 | 0.281 |
| **Vitality** | 36 | <0.001** | 22.425 | <0.001** |
| **QoL Rating** | 37 | <0.001** | 7.172 | 0.127* |
| **Health Satisfaction** | 24.8 | <0.001** | 18.741 | <0.001** |
| **General Health and QoL** | 92.4 | <0.001** | 14.425 | <0.001** |
| **Health Rating** | 56.5 | <0.001** | 26.544 | <0.001** |
| **Health comparison to 1 year ago** | 31.4 | <0.001** | 17.345 | 0.002** |
| **Personal Factors** | 82.3 | <0.001** | 14.503 | <0.001** |

* p<0.2

**p<0.05

**Supplement SC. Sociodemographic characteristics of ‘Exercisers’, ‘Non-exercisers’, ‘Guideline-adherents’ and ‘Non-adherents’ (Australia, 2022).**

|  | **Exercisers (n=910)** | **Non-exercisers (n=669)** | **Exercisers who hit guidelines (n=204)** | **Exercisers who don’t hit guidelines (n=706)** |
| --- | --- | --- | --- | --- |
| Sociodemographic | Freq (%), Mean (±SD), Min \| Max | Freq (%), Mean (±SD) Min \| Max | Freq (%), Mean (±SD) Min \| Max | Freq (%), Mean (±SD) Min \| Max |
| **Sex** | | | | |
| Responses | 910 (100) | 669 (100) | 204 (100) | 706 (100) |
| Missing | 0 (0) | 0 (0) | 0 (0) | 0 (0) |
| Male | 672 (73.8) | 485 (72.5) | 160 (78) | 512 (72.5) |
| Female | 238 (26.2) | 184 (27.5) | 44 (22) | 194 (27.5) |
| **Age** | | |  |  |
| Responses | 909 (99.9), 57 (±14) yr old,19\|94 yr old | 668 (99.9), 59 (±14) yr old, 21\|93 yr old | 204 (100), 55 (±15) yr old ,19\|83 yr old | 705 (99.9), 57 (±14) yr old, 20\|94 yr old |
| Missing | 1 (0.1) | 1 (0.1) | 0 (0) | 1 (0.1) |
| **Injury Level and Completeness** | | | | |
| Responses | 864 (94.9) | 617 (92.2) | 195 (95.6) | 669 (94.8) |
| Missing | 46 (5.1) | 52 (7.8) | 9 (4.4) | 37 (5.2) |
| Incomplete Paraplegia | 310 (34.1) | 232 (34.7) | 74 (36.3) | 236 (33.4) |
| Complete Paraplegia | 211 (23.2) | 151 (22.6) | 48 (23.5) | 163 (23.1) |
| Incomplete Tetraplegia | 274 (30.1) | 175 (26.2) | 61 (29.9) | 213 (23.2) |
| Complete Tetraplegia | 69 (7.6) | 59 (8.8) | 12 (5.9) | 57 (8.1) |
| **Time since Injury** | | | | |
| Responses | 898 (98.7), 16 (±13) yrs, 1\|59 yrs, | 644 (96.3), 19 (±15) yrs, 1\|73 yrs, | 202 (99), 15 (±13) yrs, 1\|53 yrs | 696 (98.6), 16 (±13) yrs, 1\|59 yrs |
| Missing | 12 (1.3) | 25 (3.7) | 2 (1) | 10 (1.4) |
| **Cause of Injury** | | | | |
| Responses | 910 (100) | 669 (100) | 208 (100) | 706 (100) |
| Missing | 0 (0) | 0 (0) | 0 (0) | 0 (0) |
| Traumatic | 771 (84.7) | 535 (80) | 183 (89.7) | 588 (83.3) |
| Non-traumatic | 134 (14.7) | 123 (18.4) | 19 (9.3) | 115 (16.3) |
| Unknown | 5 (0.5) | 11 (1.6) | 2 (1) | 3 (0.4) |
| **Marriage Status** | | | | |
| Responses | 909 (99.9) | 665 (99.4) | 203 (99.7) | 701 (99.9) |
| Missing | 1 (0.1) | 4 (0.6) | 5 (0.3) | 1 (0.1) |
| Single | 218 (24) | 168 (25.1) | 46 (22.5) | 172 (24.4) |
| Married | 478 (52.5) | 313 (46.8) | 100 (49) | 378 (53.5) |
| Widowed | 34 (3.7) | 28 (4.2) | 8 (3.9) | 26 (3.7) |
| Separated or Divorced | 97 (10.7) | 98 (14.6) | 26 (12.7) | 71 (10.1) |
| Cohabiting or Partnership | 82 (9) | 58 (8.7) | 24 (11.8) | 58 (8.2) |
| **Income for Household** | | | | |
| Responses | 826 (90.8) | 550 (82.2) | 186 (91.2) | 640 (90.7) |
| Missing | 84 (9.2) | 119 (17.8) | 18 (8.8) | 66 (9.3) |
| <$455/wk | 182 (20) | 174 (26) | 43 (21.1) | 139 (19.7) |
| $456-$686/wk | 113 (12.4) | 81 (12.1) | 21 (10.3) | 92 (13) |
| $687-909/wk | 93 (10.2) | 71 (10.6) | 13 (6.4) | 80 (11.3) |
| $910-$1,203/wk | 95 (10.4) | 51 (7.6) | 20 (9.8) | 75 (10.6) |
| $1,204-$1,548/wk | 98 (10.8) | 48 (7.2) | 25 (12.3) | 73 (10.3) |
| $1,549-$1,931/wk | 81 (8.9) | 40 (6) | 21 (10.3) | 60 (8.5) |
| $1,932-$2,374/wk | 53 (5.8) | 33 (4.9) | 10 (4.9) | 43 (6.1) |
| $2,375-$2,969/wk | 34 (3.7) | 17 (2.5) | 15 (7.4) | 19 (2.7) |
| $2,970-$3,979/wk | 40 (4.4) | 22 (3.3) | 8 (3.9) | 32 (4.5) |
| ≥$3,980/wk | 37 (4.1) | 13 (1.9) | 10 (4.9) | 27 (3.8) |
| **Employed before SCI** | | | | |
| Responses | 889 (97.7) | 648 (96.9) | 197 (96.6) | 692 (98) |
| Missing | 21 (2.3) | 21 (3.1) | 7 (3.4) | 14 (2) |
| Yes | 746 (82) | 553 (82.7) | 163 (79.9) | 583 (82.6) |
| No | 143 (15.7) | 95 (14.2) | 34 (16.7) | 109 (15.4) |
| **Employed currently** | | | | |
| Responses | 888 (97.6) | 22 (3.3) | 197 (96.6) | 691 (97.9) |
| Missing | 22 (2.4) | 647 (96.7) | 7 (3.4) | 15 (2.1) |
| Yes | 265 (29.1) | 185 (27.7) | 44 (21.6) | 221 (31.3) |
| No | 623 (68.5) | 462 (69.1) | 153 (75) | 470 (66.6) |
| **Education Level** | | | | |
| Responses | 898 (98.7) | 653 (97.6) | 198 (97.1) | 700 (99.2) |
| Missing | 12 (1.3) | 16 (2.4) | 6 (2.9) | 6 (0.8) |
| Primary | 25 (2.7) | 36 (5.4) | 6 (2.9) | 19 (2.7) |
| Lower Secondary | 216 (23.7) | 218 (32.6) | 50 (24.4) | 166 (23.5) |
| Higher Secondary | 112 (12.3) | 95 (14.2) | 23 (11.3) | 89 (12.6) |
| Post-Secondary | 176 (19.3) | 116 (17.3) | 42 (20.6) | 134 (19) |
| Short Tertiary | 102 (11.2) | 77 (11.5) | 23 (10.3) | 79 (11.2) |
| Bachelor or equivalent | 162 (17.9) | 76 (11.4) | 32 (15.7) | 131 (18.6) |
| Master or equivalent | 102 (11.2) | 35 (5.2) | 22 (10.8) | 80 (11.3) |
| Other | 2 (0.2) | 0 (0) | 0 (0) | 2 (0.3) |
| **Years of Education pre-SCI** | | | | |
| Responses | 843 (92.6), 11 (±5) yrs, 0\|30 yrs, | 581 (86.6), 10 (±5) yrs, 0\|30 yrs | 189 (92.6), 12 (±5) yrs, 0\|26 yrs | 654 (92.6), 11 (±5) yrs, 0\|30 yrs |
| Missing | 67 (7.4) | 88 (13.2) | 15 (7.4) | 52 (7.4) |
| **Years of Education post-SCI** | | |  |  |
| Responses | 732 (80.4), 2 (±3) yrs, 0\|28 yrs | 491 (73.4), 2 (±4) yrs, 0\|29 yrs | 163 (79.9), 1 (±0) yrs, 0\|16 yrs | 569 (80.6), 2 (±3) yrs, 0\|28 yrs |
| Missing | 178 (19.6) | 119 (17.8) | 41 (20.1) | 137 (19.4) |

*SD = standard deviation; SCI = spinal cord injury*

**Supplement SD. Psychosocial characteristics of ‘Exercisers’, ‘Non-exercisers’, ‘Guideline-adherents’ and ‘Non-adherents’ (Australia, 2022).**

| Psychosocial characteristic | Frequency (%) | Mean (±SD) or Median |
| --- | --- | --- |
| **Vitality** | | |
| All participants | 1,533 (97) | 49.1 (±23) |
| Exercisers | 895 (57) | 52 (±22.4) |
| Non-exercisers | 638 (40) | 45 (±23.2) |
| Guideline-adherents | 201 (22.1) | 59.2 (±21.1) |
| Guideline non-adherents | 694 (76.3) | 50 (±22.3) |
| **Quality of Life Rating** | | |
| All participants | 1,518 (96) | 4. Good |
| Exercisers | 906 (57) | 4. Good |
| Non-exercisers | 612 (39) | 4. Good |
| Guideline-adherents | 204 (22.4) | 4. Good |
| Guideline non-adherents | 702 (77.1) | 4. Good |
| **Health** **Satisfaction** | | |
| All participants | 1,519 (96) | 3. Neither satisfied nor dissatisfied |
| Exercisers | 907 (57) | 4. Satisfied |
| Non-exercisers | 612 (39) | 3. Neither satisfied nor dissatisfied |
| Guideline-adherents | 203 (22.3) | 4. Satisfied |
| Guideline non-adherents | 704 (77.4) | 3. Neither satisfied nor dissatisfied |
| **General Health and Quality of Life** | | |
| All participants | 1,578 (99.9) | 13.9 (±4.2) |
| Exercisers | 910 (58) | 14.8 (±3.2) |
| Non-exercisers | 668 (42) | 12.7 (±5.2) |
| Guideline-adherents | 204 (22.4) | 15.5 (±3) |
| Guideline non-adherents | 706 (77.6) | 14.5 (±3.2) |
| **Health Rating** |  | |
| All participants | 1,513 (96) | 3. Good |
| Exercisers | 907 (57) | 3. Good |
| Non-exercisers | 606 (38) | 3. Good |
| Guideline-adherents | 202 (22.2) | 3. Good |
| Guideline non-adherents | 705 (77.5) | 3. Good |
| **Health Comparison to 1 year ago** | | |
| All participants | 1,517 (96) | 3. About the same |
| Exercisers | 909 (58) | 3. About the same |
| Non-exercisers | 608 (39) | 3. About the same |
| Guideline-adherents | 203 (22.3) | 3. About the same |
| Guideline non-adherents | 706 (77.6) | 3. About the same |
| **Personal Factors** | | |
| All participants | 1,579 (100) | 14 (±4.4) |
| Exercisers | 910 (58) | 14.8 (±3.4) |
| Non-exercisers | 669 (42) | 12.8 (±5.3) |
| Guideline-adherents | 204 (22.4) | 15.6 (±3.2) |
| Guideline non-adherents | 706 (77.6) | 14.6 (±3.5) |

*SD = standard deviation*
